# Editorial: Advances in treatment planning, optimization and delivery for radiotherapy of breast cancer

**DOI:** 10.3389/fonc.2023.1354731

**Published:** 2024-01-08

**Authors:** Vishruta Dumane, Nisha Ohri, Jehee Isabelle Choi, Arpit Chhabra, Haibo Lin

**Affiliations:** ^1^ Department of Radiation Oncology, Icahn School of Medicine at Mount Sinai, New York, NY, United States; ^2^ Department of Radiation Oncology, Rutgers Cancer Institute of New Jersey, New Brunswick, NJ, United States; ^3^ Department of Radiation Oncology, New York Proton Center, New York, NY, United States

**Keywords:** breast cancer, hypo-fractionation, partial breast irradiation, post-mastectomy radiation therapy, deep inspiration breath-hold, knowledge-based planning, volumetric modulated arc therapy, proton therapy

## Introduction

Various planning, optimization, delivery, and treatment modalities, as well as their fractionations, are constantly being investigated or updated to improve the therapeutic ratio for breast cancer patient care. Advanced treatment planning and delivery methods, such as volumetric modulated arc therapy (VMAT), have been applied in complex anatomical scenarios where standard 3D conformal planning techniques have failed (1). Although VMAT is useful in these situations, long-term follow-up data on toxicity due to low-dose exposure to VMAT is relatively scarce. Knowledge-based planning (KBP) has been applied to predict optimally achievable dose distributions in a given patient anatomy and to determine if a specific delivery technique is suitable for a patient, minimizing the likelihood of toxicity (2). Moderate hypofractionation decreases the logistic burden and cost to patients and healthcare systems, making it the standard of care for whole breast irradiation (WBI) (3–5). However, there is a paucity of data on toxicity when using moderate hypofractionation for regional nodal irradiation (RNI). Minimizing treatment volume from whole breast to partial breast irradiation (PBI), in appropriately selected patients, has further helped improve patient outcomes, thus supporting its use (6, 7). However, deploying PBI among a larger patient population that can benefit from it is an area of active investigation in the community. The dosimetric advantages of proton radiation for breast cancer are well established (8), and its use in the postmastectomy radiation therapy (PMRT) setting with either tissue expander or implant reconstruction is increasing. However, more data are needed when it comes to implant safety and toxicity with proton therapy. The potential application of MR-guided radiation therapy (MRgRT) as a neoadjuvant therapy to shrink tumor volume has been discussed for PBI (9). An improved understanding of the quantification and dosimetric impact of the electron stream effect (ESE) will only help increase its use for WBI and PMRT with RNI while simultaneously improving tumor visualization. Our Research Topic titled “*Advances in Treatment Planning, Optimization, and Delivery for Radiotherapy of Breast Cancer*” is dedicated to featuring original research and review articles addressing some of these topics and the potential paucity of data in these areas.

## Topics covered in this editorial

Knowledge-based planning, delivery, and dosimetry for breast cancer: Phurailatpam et al., Quesada et al., Li et al., Prunaretty et al., and Ramos-Mendez et al.

Hypofractionation and axillary nodal irradiation in breast cancer: Chitapanarux et al. and Elumalai et al.

Partial breast radiation: Le et al., Rhome et al., and Galavis et al.

Proton therapy for breast cancer: Chen et al. and Sayan et al.

MRI-guided radiotherapy for breast cancer: Lee et al.

## Articles included in this Research Topic

Many patients in low- and middle-income countries (LMICs) present with locally advanced disease, requiring PMRT and RNI as part of their adjuvant treatment. Phurailatpam et al. designed an efficient workflow using VMAT and KBP for moderate and ultra-hypofractionation for these patients. The automated plans were less complex, improving the efficiency of treatment delivery and impacting the workflow in a busy clinic, thus amalgamating KBP in a decreasing treatment planning burden while planning for patients requiring RNI with hypofractionation.

The need to irradiate IMNs increases heart and lung exposure, and VMAT is known to reduce the dose for these while generating more conformal isodose distributions (1, 10). Quesada et al. addressed the feasibility of VMAT in the treatment of bilateral breast with regional nodes. They reported on long-term follow-up concerning the toxicity and safety of VMAT for the largest cohort of patients in this setting.

Deep inspiration breath-hold (DIBH) reduces the extent of low-dose exposure to normal tissue (10). Li et al. investigated tangent-based arcs to further improve dosimetry over partial VMAT using DIBH. This significantly reduced treatment time, making the treatment more clinically viable.

During treatment planning, factoring surrogates that are predictors of late toxicity is essential. Although such surrogates are reliable for cardiac toxicities in conventional planning, their understanding of advanced planning such as VMAT is scarce. Prunaretty et al. investigated this for left-sided breast cancer patients with unfavorable cardiac anatomy requiring IMRT/VMAT for improved sparing, and they concluded that a heart volume receiving dose ≥ 40 Gy is a better surrogate.

With the increasing use of tissue expanders in the postmastectomy setting, the safety and accuracy of dose calculation in these cases cannot be overemphasized. Ramos-Mendez et al. presented the first comprehensive evaluation of treatment planning strategies accounting for artifacts introduced by tissue expanders and verified it via Monte Carlo calculations, the collapsed cone dose calculation algorithm, and measurement with film. The highest discrepancies in the calculations in their study were noted when artifacts were assumed to have the dosimetric properties of water. These errors could be reduced if the tissue expander geometry and materials were used instead.

Patient eligibility to safely receive PBI is sensitive to when the CT scan is performed for treatment planning. Le et al. first reported the impact of factors other than time post-surgery on the healing of the cavity in the postoperative period, such as body mass index, receipt of neoadjuvant chemotherapy, hypertension, and patient positioning, serving as a reference for safe delivery of PBI.

Triple-negative breast cancer (TNBC) has inferior overall survival, disease-free survival, and local control. The use of PBI can potentially help reduce toxicity over WBI (current standard of care for TNBC) in the concurrent setting while improving logistics. Rhome et al. reported on the outcomes of patients with TNBC treated prospectively with post-lumpectomy PBI and concurrent chemotherapy compared with a matched WBI cohort. The promising results presented in this study are hypothesis generating for prospective clinical trials.

Galavis et al. discussed the PBI delivery technique and the current trends in research to help better define patient selection, treatment delivery, treatment planning dosimetry, and outcomes with respect to toxicity.

There is a relative paucity of data on toxicity profiles for patients receiving regional nodal irradiation (RNI) with hypofractionation and simultaneous integrated boost (SIB). Chitapanarux et al. reported acute toxicities with respect to skin and hematologic function for patients receiving hypofractionation prospectively with helical tomotherapy to the intact breast and regional lymph nodes after BCS and adjuvant chemotherapy. The results were acceptable in both endpoints.

With studies maturing on the use of hypofractionation in the RNI setting, Elumalai et al. presented the latest guidelines and evidence on the management of the axilla with surgery versus radiation.

Chen et al. presented a case study to show the dosimetric impact of a dislocated metallic port of a breast tissue expander while receiving proton therapy and its impact on cumulative dose due to its potential dislocations during treatment.

With the increasing use of proton therapy in post-mastectomy, more data are needed on its use in the reconstruction setting. Sayan et al. presented a retrospective comparison of acute toxicities and reconstructive complications in patients treated with proton-based and photon-based PMRT. They concluded that acute skin toxicity was the most frequent adverse event in PMRT for both modalities. Reconstructive complications were not significantly higher with proton therapy.

Lee et al. quantified the dosimetric impact of the electron stream effect (ESE) during 0.35T MRgRT, along with a discussion on how these excess doses due to ESE can be reduced and the implications for treatment planning after BCS or mastectomy.

## Conclusions and future outlook

As results from randomized clinical trials such FABREC are being reported, while the RT CHARM is to arrive within the next year, there are likely to be more and more patients receiving RNI in the PMRT setting, increasing the likelihood of complex anatomies treated with hypofractionation. The need to meet coverage constraints, conformity, and homogeneity while sparing normal tissue from low doses will necessitate deploying these advanced planning, optimization, and delivery methods, such as VMAT and DIBH, while emphasizing new treatment modalities, such as protons. The SHARE trial being made available, which confirms the non-inferiority of APBI to WBI, will also encourage the increased use of the former in the treatment of select patients. With improved image guidance and real-time tumor visualization with MRgRT, the therapeutic ratio is likely to be further enhanced. We hope that through these diverse arrays of topics covering original research and review articles, we have addressed some of the scarcity in the data in a way that could potentially be supplementary and useful in further supporting the safe and efficacious use of these treatments and planning and delivery methods.

## Author contributions

VD: Writing – original draft, Writing – review & editing. NO: Writing – review & editing. JC: Writing – review & editing. AC: Writing – review & editing. HL: Writing – review & editing.
